# Retrospective cohort study analyzing temporal bone cortical thickness and perioperative complication rate, in pediatric cochlear implantation

**DOI:** 10.3389/fped.2025.1665266

**Published:** 2025-10-31

**Authors:** Laura M. Markodimitraki, Jan W. Dankbaar, Inge Stegeman, Hans G. X. M. Thomeer

**Affiliations:** ^1^ Department of Otorhinolaryngology and Head & Neck Surgery, University Medical Center Utrecht, Utrecht University, Utrecht, Netherlands; ^2^ UMC Utrecht Brain Center, Utrecht University, Utrecht, Netherlands; ^3^ Department of Radiology and Nuclear Medicine, University Medical Center Utrecht, Utrecht, Netherlands

**Keywords:** cochlear implant, surgery, complications, device failure, pediatric

## Abstract

**Background:**

Cochlear implant fixation in pediatric patients can be challenging due to the thin cranial bone. The dura matter can be exposed by drilling a bony recess leading to possible complications. A minimally invasive newer fixation method might avoid such risks.

**Objectives:**

The study focus is to assess the feasibility of drilling a bony well adequate for cochlear implant receiver/stimulator device embedment in pediatric patients of different age groups. We also aim report the occurred complications and device failure rates using different surgical techniques for cochlear approach and fixation of the implant.

**Methods:**

Computed tomography (CT) scans of 96 pediatric patients (192 ears) were acquired. An optimal location was found within a predetermined area of the temporal bone, using an in-house designed algorithm in Materialise Python API. The feasibility of drilling a bony well was assessed by digitally removing a ramped shaped bony well. Skull thickness descriptive data were calculated, before and after the removal of the bone. Clinical data of pediatric CI patients receiving their cochlear implant between 1996 and 2021 in our tertiary center, were retrospectively collected.

**Results:**

In 153 ears (79.7%) it was not feasible to create a bony well without exposing the dura mater. In young children aged 0-4 years, drilling a bony well was not feasible in almost all patients (n=69, 98.6%). Mean minimum bone thickness of the location determined by the algorithm, in different age groups, varied from 1.84 mm in the 0-4 years, to 3.31 mm in the 15-17 years age group. We included 344 cochlear implants in 230 patients with a mean age of 3 years. Most implants were placed using the mastoidectomy with posterior tympanotomy (MPTA) approach technique (n=256, 74.4%) and fixated with the bony well fixation technique with or without bony tie-down sutures (n=293, 85.1%). Major complications occurred in all surgical techniques groups. Device related complications occurred in both the bony well and the tight pocket groups.

**Conclusion:**

Drilling a bony well for fixation of the cochlear implant without exposing the dura matter is not feaible in children. No difference in complication rates was reported regarding device failure between subgroups.

## 1 Introduction

For infants and children with severe to profound sensorineural hearing loss, either congenital or acquired, cochlear implantation has become standard care. Literature shows that implantation in pediatric patients at early age is beneficial for auditory development, and minimizes language delays that result from hearing loss (1, 2). Bilateral cochlear implantation (binaural stimulation) in children provides even more benefits, leads to increased audiophysiological stimulation of the auditory cortex at an early age, and is therefore the mainstay of treatment in children that meet implantation criteria in Dutch healthcare (3, 4).

Cochlear implantation surgery has been proven to be a safe procedure, with low complication rates. Revision surgery rates vary between 4.6% and 8.7%, and are mostly due to device failure as a result of which re-implantation is necessary (5–8). Complications that are not due to device failure, such as migration or protrusion of the receiver/stimulator (R/S) device, wound infection with implant extrusion or electrode misplacement or migration can also occur (5, 9–12). The complication rate reported in the literature varies greatly between studies with reported rates of 0.6% to 30.9% (9, 11–14). Due to broadening of the indication criteria and expected improved functional outcome after bilateral cochlear implantation, more children are receiving a CI and at a younger age (3, 15).

Recent publications stress the importance of recognizing the challenges associated with operating on young children in order to prevent complications (2, 16). The standard surgical technique for cochlear implantation in our center is the mastoidectomy with posterior tympanotomy approach (MPTA). The alternative suprameatal approach (SMA) has also been used, although Bruijnzeel et al. (14) reported a higher (infectious) complication rate when using this technique. It would be informative to update and assess these data with a prolonged follow up. Another important step in the surgical procedure is the positioning and fixation of the R/S device which can be achieved by several surgical techniques. The most used bony well technique, requires drilling a recess in the temporal bone in which the implant will reside. Usually a canal, tunnel or overhang is made for protection of the electrode array. Some CI surgeons use additional sutures, screws or wires to secure the implant. The less invasive subperiosteal pocket technique uses the soft tissue of the pericranium-temporalis muscle to fixate the implant (17). Both techniques of CI fixation have been used in our academic medical center over the years since the start of pediatric cochlear implantation in 1996. However, our experience is that drilling a bony well to accommodate the implant is not always feasible in young children due to insufficient skull thickness. Cochlear implant manufacturers advise a bony recess depth of at least 1.0–3.0 mm for sufficient fixation of the device, depending on the implant model (18–20). In order to lower the profile of the housing, an even deeper recess is required. This is challenging in infants, with their immature skull thickness. Furthermore, the dimensions of a cochlear implant demand a bony recess with a width of at least 30 mm to house the case. The curvature and irregularity of the temporal bone make embedment of a flat surface such as a CI challenging. Additionally, attempting drilling a bony well without preoperative imaging data or planning to measure thickness, introduces possible risks to the patient. However, these attempts of drilling would be redundant if we knew that drilling a bony well under a certain age is not feasible or necessary.

Previous studies describe an adaptation of the fixation technique where (partial) exposure of the dura is necessary and a bony island is left in the center to function as resistant and protective layer (21–23). These studies demonstrate the difficulty of drilling in young infants and the risks involved. Possible complications associated with drilling are dural tears with subsequent cerebrospinal fluid leakage as a direct result of drilling close to the dura (10, 21, 24). Other complications that have been reported (but occur very rarely) and associated with the bony well technique are late onset hematomas, epi-/subdural hematoma, tentorial herniation, and cerebral infarction, as well as meningitis (24–29).

Therefore, in this study we aim to assess the feasibility of drilling a bony well adequate for CI embedment in different age groups. We also aim to review the pediatric cohort implanted in our institution, reporting the occurred complications, revision and device failure rate using different surgical techniques.

## 2 Materials and methods

This mono-center, retrospective and exploratory study was conducted at the University Medical Centre (UMC) Utrecht The Netherlands, in compliance with the principles of the Declaration of Helsinki. Exemption was granted by the local ethical committee (Institutional Review Board of the UMC Utrecht) (METC protocol 22/560) as a non-WMO study where consent was provided. The exemption included the CT data as well as clinical cohort data. All data was pseudonymized, thus exempt from acquiring informed consent.

### 2.1 Imaging data collection and analysis

**Figure 1:**
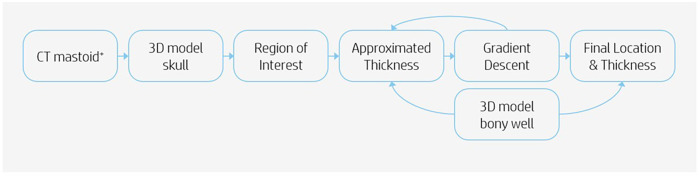
Flowchart of CT scan analysis.

**Figure 2:**
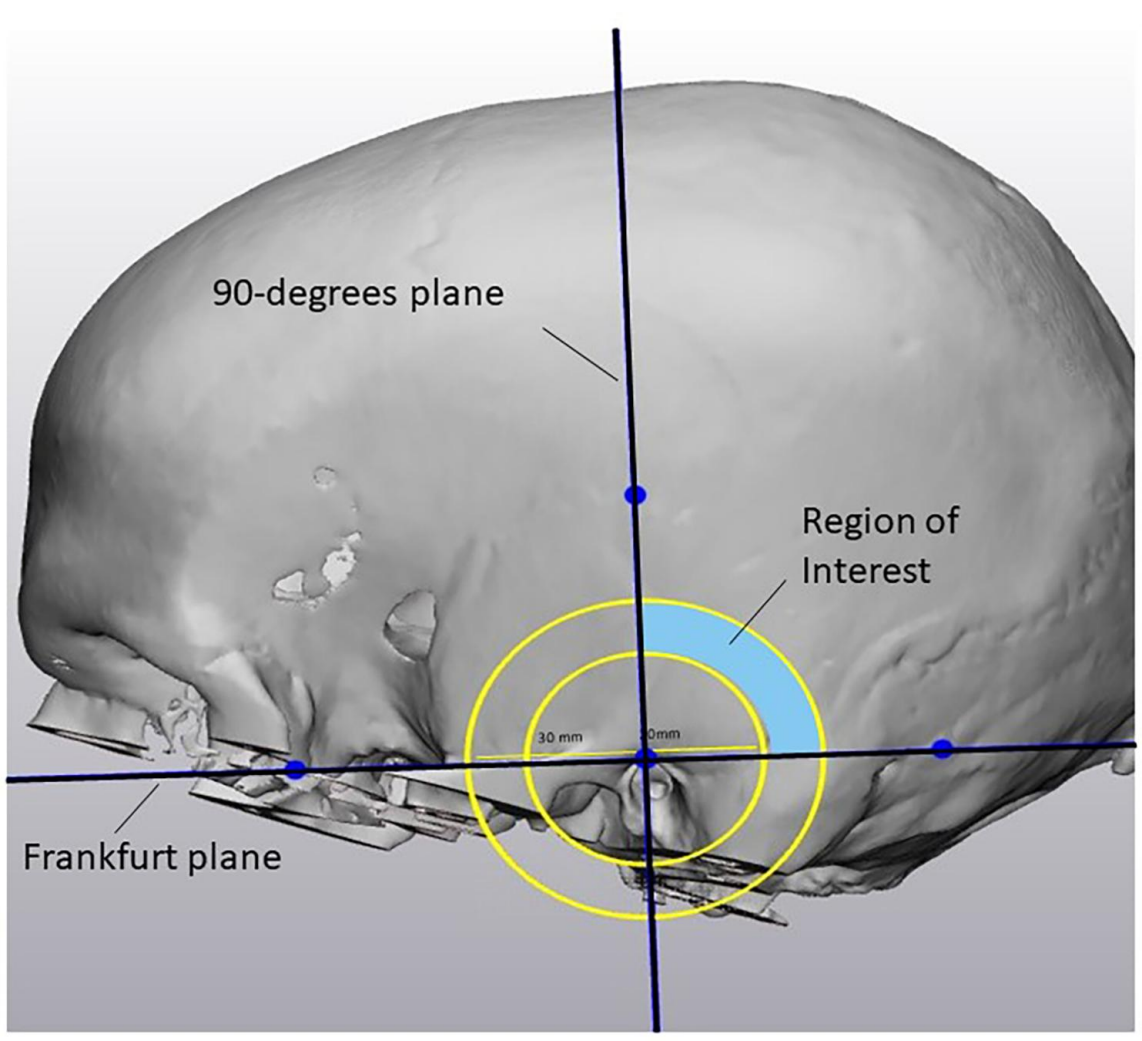
Boundaries of the region of interest.

Imaging data analysis was realized using computed tomography (CT) scans of 96 pediatric patients. These scans included the temporal bone bilaterally. Each ear was seen as an individual case. The indications for the scans were not considered. The pseudonymized CT scans were identified via the appropriate radiologic code, made available for research. The information of the temporal bone thickness was analyzed as follows. Scans were imported into the software program Mimics (version 24.0, Materialise NV, Leuven, Belgium) for segmentation of the scan. A 3D model of the skull was exported in Materialise 3-matic (version 16.0, Materialise. Leuven, Belgium). To determine if it was feasible to drill out a bony well, an in-house developed script was used to automate the analysis. The automation was done using Python scripting and the Materialise Python API. The analysis was performed based on the following steps (Figure 1). Firstly, not each location on the temporal bone is suitable for placement of the R/S device. Therefore a region on each skull was determined in which the feasibility analysis took place, defined as the region of interest (ROI). The boundaries of this region were the following: the Frankfurter Horizontal plane, a perpendicular plane originating from the external auditory canal (EAC), a minimum radius of 20 mm from the EAC and a maximum radius of 30 mm from the EAC (Figure 2). Secondly, a systematic search must be performed within the ROI to identify the location in which the cortical thickness would be sufficient to implant the CI. This was realized using a gradient descent algorithm that approximates the gradient of the skull thickness determined by the size and location of the bony well. Thirdly, a 3D model of the bony well was used to subtract digitally from the ROI. This 3D model was ramped shaped, based on the dimensions of the Cochlear CI512 model. A thickness of 5.0 mm at the anterior edge of the bony well was used. Feasibility of drilling a bony well was determined based on the remaining skull thickness after digital removal of the bony well. The remaining skull had to be intact. Skull thickness descriptive data were calculated for the specific area where the bony well was digitally made, before and after the removal of the bone.

### 2.2 Clinical data review

A retrospective chart review of pediatric patients who underwent primary cochlear implant surgery in our center between January 1, 1996 and December 31, 2021 was conducted. These patients were identified from the electronic patient dossier with the code of the surgical procedure. All patients that were younger than 18 years of age at the time of implantation were included. Patients were excluded if the postoperative follow up was less than 12 months. Each operated ear was considered an individual case. Calculations per case were performed to overcome between-patient variability (in bilateral cases). Clinical data were reviewed to collect demographic records, the date of the first implant, the surgical techniques used for cochlear approach and fixation of the CI, the type of CI, complications and device failures. The primary endpoint of the study is the complication rate per ear in this study. Secondary endpoints such as R/S device-related issues or device failure are assessed. In our cohort both the mastoidectomy with posterior tympanotomy approach (MPTA) and the suprameatal approach (SMA) techniques were used for cochlear implantation. For the fixation of the R/S device the applied surgical techniques include drilling a bony well with or without tie-down sutures, and the minimally invasive subperiosteal tight pocket technique. Complications were classified into major and minor according to the proposal of Hansen et al. (30), and into peri- and postoperative depending on the time of presentation. Perioperative complications include complications occurring during and up to 24 h after surgery. Pre-existing conditions were not classified as a complication if encountered postoperatively. Cases in which revision surgery took place, causative mechanisms for revision such as device failure and the time between operation and revision were reported. Device failure was classified into hard or soft failure using the standardized criteria described in the 2005 in the Cochlear Implant Soft Failures Consensus Development Conference Statement (31). This report follows the STROBE guidelines for cohort studies (Supplementary Materials).

## 3 Results

### 3.1 CT data analysis

**Figure 3:**
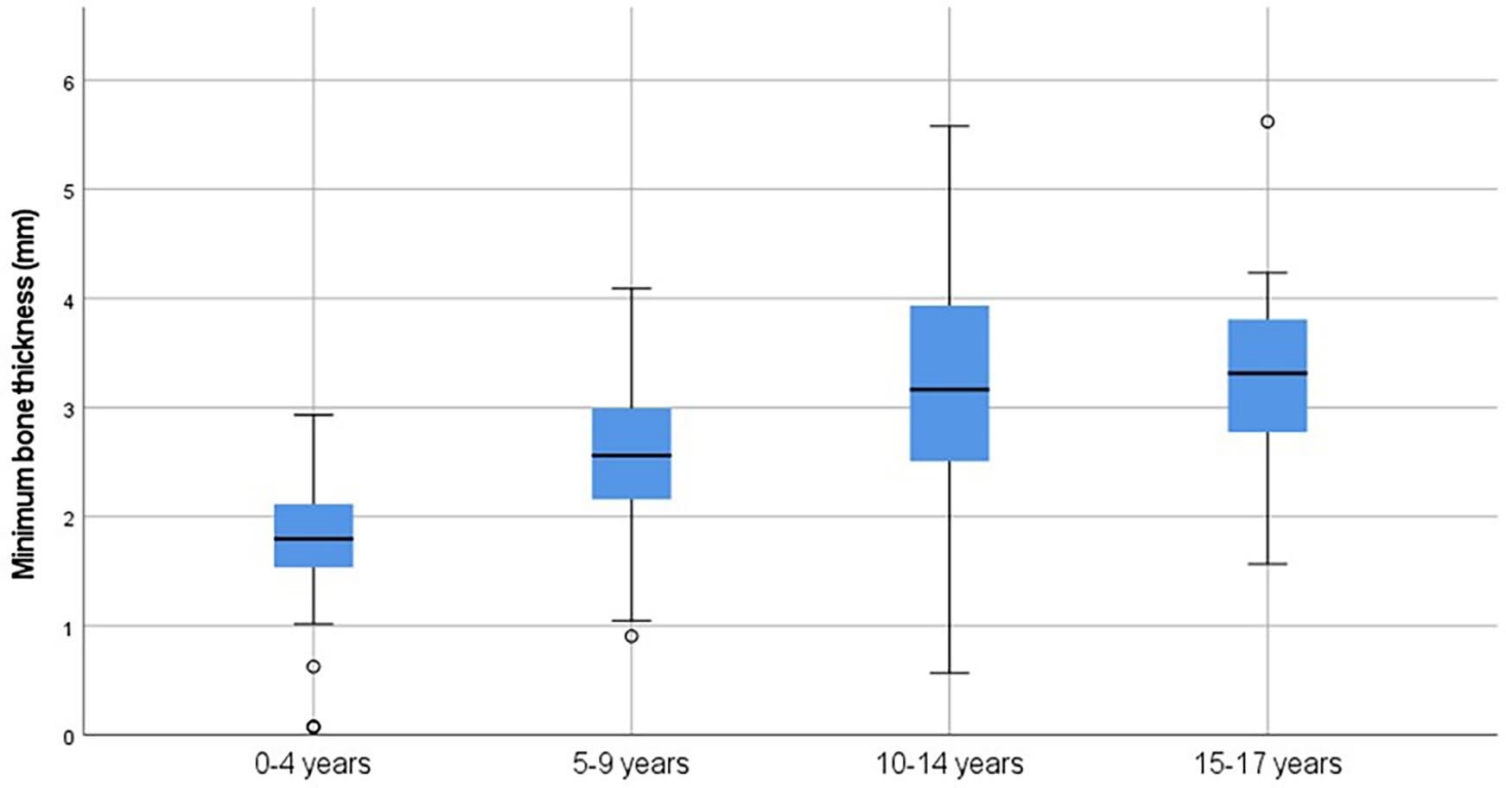
Minimum bone thickness per age group.

Most ears analyzed were from male patients (*n* = 118, 61.5%). The largest age group was zero to four years of age (*n* = 70, 36.5%)(Table 1). In the majority of the analyzed ears, it was not feasible to drill a bony well (*n* = 153, 79.7%), meaning that the remaining skull after digital removal of the bony well was not intact in these cases (see methods section). This was especially frequent in the zero to four age group (*n* = 69, 98.6%). We found that the minimum bone thickness in all cases in this age group was below 3 mm (Figure 3). As expected, the number of cases in which it was feasible to drill a bony well increased per age group (Table 2).

**Table 1:** CT-scan data analysis.

Ears scanned (*n* = 192)	N	%
Gender		
Male	118	61.5%
Female	74	38.5%
Age groups		
0 to 4 years	70	36.5%
5 to 9 years	44	22.9%
10 to 14 years	52	27.1%
15 to 17 years	26	13.5%
Feasibility of drilling a bony well		
Not feasible	153	79.7%
Feasible	39	20.3%

**Table 2:** CT-scan data analysis per age group.

Ears scanned (*n* = 192)	0–4 years	5–9 years	10–14 years	15–17 years
Feasibility of drilling a bony well *n* (%)				
Not feasible	69 (98.6%)	38 (86.4%)	35 (67.3%)	11 (42.3%)
Feasible	1 (1.4%)	6 (13.6%)	17 (32.7%)	15 (57.7%)
Minimum bone thickness (mm)				
Mean (SD)	1.84 (0.57)	2.58 (0.78)	3.10 (1.03)	3.31 (0.77)
Min	0.07	0.91	0.57	1.57
Max	2.93	4.09	5.58	5.62

### 3.2 Demographics of clinical data

We identified 383 implanted ears, 39 were excluded due to lack of information (*n* = 4), follow up of <12 months (*n* = 32), three cases were operated in a different medical center. A total of 344 ears of 230 patients were included in our study (Table 3). Ages ranged from 4 months in a child with SNHL after meningitis, to 18 years and 6 months, with a mean age of 3 years and 7 months at time of surgery. The majority of the cases (*N* = 229, 66.6%) were bilaterally implanted, of which 132 cases (57.6%) simultaneously. One patient was included as bilaterally implanted, but the first operation took place elsewhere. The unilateral implants were placed in 73 right and 42 left ears. Most CI's implanted were Cochlear Nucleus® devices (89.2%) followed by Med-el® (6.4%) and Advanced Bionics® (4.1%). Median follow up time was 8 years and 8 months.

**Table 3:** Demographics and clinical characteristics by implant.

Implants placed (*n* = 344)	n	%
Bilateral	229	66.6%
Bilateral Sequential	97	28.2%
Bilateral Simultaneous	132	38.4%
Unilateral	115	33.4%
Placement		
Right ear	73	21.2%
Left ear	42	12.2%
Etiology		
Congenital	185	53.8%
Meningitis	58	16.9%
Genetic condition	55	16.0%
Cytomegalovirus infection	22	6.4%
Other	20	5.8%
Missing	4	1.2%
Brand of implant		
Cochlear	307	89.2%
Med-el	23	6.7%
Advanced Bionics	14	4.1%
Time of Implant Follow-up Median (range;SD)	8 y and 8 m	12 m – 25.7y;6 y

Y, years; m, months.

### 3.3 Major complications

The patient records revealed 29 major complications in 29 implanted ears (26 patients); yielding a complication rate of 8.4% per implanted ear (Tables 4, 5). Two bilaterally implanted patients had a major complications on each of their implants. One unilaterally implanted patient underwent revision surgery twice due to an incorrect electrode position, replacing the implant both times. Almost all complications were postoperative (*N* = 28, 96.6%)(Table 5). The most frequent major complication was infection at the operation site or the implant itself (*N* = 6, 20.7%), followed by electrode array migration (*N* = 5, 17.2%) and non-iatrogenic trauma (*N* = 5, 17.2%) (Table 5).

**Table 4:** Complications and device failure characteristics per implanted ear.

	Total implants *n* (%)	Major complication *n* (%)	Minor complication *n* (%)	Device failure *n* (%)
Implants placed	344 (100%)	29 (8.4%)	166 (48.3%)	16 (4.7%)
Mean age at implantation (range;SD)	3y and 7m (4 m – 18.5y;4.1y)			
Mean time to complication or failure (range;SD)¤		3y and 1m (1 m – 14y; 4.1y)	2 y and 5 m (0.3 m – 14.7y; 3.5y)	3 y and 8 m (2 m – 12.6y; 3.2y)
Brand of Implant				
Cochlear	307 (89.2%)	27 (93.1%)	146 (88.0%)	10 (62.5%)
Med-el	23 (6.7%)	1 (3.4%)	11 (6.6%)	3 (18.8%)
Advanced Bionics	14 (4.1%)	1 (3.4%)	9 (5.4%)	3 (18.8%)
Cochlea approach				
MPTA	256 (74.4%)	12 (41.4%)	127 (76.5%)	12 (75.0%)
SMA	85 (24.7%)	17 (58.6%)	37 (22.3%)	4 (25.0%)
Missing	3 (0.9%)		2 (1.2%)	
Fixation technique				
Bony well (with bony canal or tunnel)	283 (82.2%)	19 (65.4%)	136 (82.0%)	12 (75.0%)
Tight pocket	39 (11.3%)	7 (24.1%)	13 (7.8%)	3 (18.8%)
Bony well with bony sutures	10 (2.9%)	2 (6.9%)	9 (5.4%)	1 (6.3%)
Unknown/Missing	12 (3.5%)	1 (3.4%)	8 (4.8%)	
User or non-user				
User	316 (91.9%)	22 (75.9%)	150 (90.4%)	14 (87.5%)
Non-user	26 (7.6%)	7 (24.1%)	16 (9.6%)	2 (12.5%)
Missing	2 (0.6%)			

Y, years; m, months.

Time to major or minor complication were calculated per implant in years only for the postoperative complications. Perioperative were not taken into account.

**Table 5:** Major complications characteristics .

	Major complications n (%)
Time to complication	
Perioperative	1 (3.4%)
Postoperative	28 (96.6%)
Perioperative complications	
Tip foldover	1 (3.4%)
Postoperative complications	
Infection of operation site or implant site	6 (20.7%)
Electrode array migration	5 (17.2%)
Non-iatrogenic trauma	5 (17.2%)
Electrode array issue	3 (10.4%)
R/S migration	2 (6.9%)
Pain at operation site or implant site	2 (6.9%)
Facial nerve paralysis	1 (3.4%)
Mastoiditis	1 (3.4%)
Magnet related issues	1 (3.4%)
Facial nerve stimulation	1 (3.4%)
Cholesteatoma	1 (3.4%)
Treatment of complications	
Revision surgery	18 (62.1%)
Explantation	9 (31%)
Minor surgical procedure	1 (3.4%)
Hospitalization for treatment	1 (3.4%)
Total	29 (100%)

The majority of cases with major complications were operated with the SMA surgical technique (*N* = 17, 58.6%). The tight pocket technique was used more frequently (*N* = 7, 24.1%) in the major complications subgroup than the general cohort (*N* = 39, 11.3%). All cases operated with the tight pocket technique, were also operated with the SMA technique. Most major complications required revision surgery (*N* = 18, 62.1%); nine cases had to be explanted (31%). In five cases, the patients did not receive a new implant after explantation. One of these five patients was bilaterally implanted and had major complications in both ears, permanent facial nerve paralysis and infection of the implant. This patient deceased due to a pre-existing neurological condition. One bilaterally implanted patient became a non-user of the left ear due to magnet problems (the magnet falling off the head) despite conservative and invasive attempts to elevate the issue.

### 3.4 Minor complications

We reported 227 minor complications in 166 implants (132 patients), yielding a complication rate of 48.3% per implant (Table 6). The majority of ears (114/166) was bilaterally implanted. Of those ears, 34 patients had minor complications in both ears, 46 patients had a complication only in one ear. The most frequent of the 52 (22.9%) perioperative complications, was dural exposure or tear (*N* = 13, 5.7%), followed by exposure of the facial nerve during operation (*N* = 9, 4.0%) and chorda tympani manipulation or sacrifice ((*N* = 8, 3.5%). Otitis media acuta was the most frequent postoperative complication (*N* = 51, 22.5%), followed by infection of operation site or implant (*N* = 16, 7.0%) and otitis media with effusion (*N* = 12, 5.3%). Two of the three patients that presented with R/S device migration, had been operated using the tight pocket fixation technique.

**Table 6:** Minor complications characteristics per complication.

	Minor complications n (%)
Time to complication	
Perioperative	52 (22.9%)
Postoperative	175 (77.1%)
Perioperative complications	
Dural exposure or tear	13 (5.7%)
Exposure of facial nerve during operation	9 (4.0%)
Chorda tympani manipulation or sacrifice	8 (3.5%)
Partial insertion of the electrode array	6 (2.6%)
Other iatrogenic defect during surgery	5 (2.2%)
Incus resection during surgery	5 (2.2%)
CSF leak or gusher	4 (1.8%)
Hematoma	1 (0.4%)
Other	1 (0.4%)
Postoperative complications	
(Recurrent) otitis media acuta	51 (22.5%)
Infection of operation site or implant site	16 (7.0%)
Other	18 (8.0%)
Otitis media with effusion	12 (5.3%)
Otorrhea	9 (4.0%)
Otitis externa	8 (3.5%)
Hematoma	7 (3.1%)
Mastoiditis	7 (3.1%)
Pain at operation site or implant site	6 (2.6%)
Magnet related issues	6 (2.6%)
Pain at the site of the R/S device	5 (2.2%)
Dizziness	5 (2.2%)
Non-iatrogenic trauma	4 (1.8%)
Facial oedema	4 (1.8%)
R/S migration	3 (1.3%)
Facial nerve stimulation	3 (1.3%)
R/S device issues	2 (0.9%)
Facial nerve weakness	2 (0.9%)
Meningitis	2 (0.9%)
Headache	2 (0.9%)
Pain n.o.s.	2 (0.9%)
Pain on stimulation	1 (0.4%)
Treatment of complication	
Oral or topical treatment	87 (38.3%)
Conservative treatment	56 (24.7%)
Wait and see	38 (16.7%)
Minor surgical procedure	25 (11%)
Hospitalization for treatment	20 (8.8%)
Missing	1 (0.4%)
Total	227

### 3.5 Device failures

Device failure occurred in 16 cases (4.7%), of which 14 (4.1%) were hard failures and 2 were soft failures (0.6%). Most hard failures (9/14) had no identifiable cause, in three cases the implant was defective due to trauma. In one case of electrode array migration and one case of implant infection, the devices were found to be defective after explantation. In two cases of soft failure, one case was due to unbearable pain at the implant site due to which the implant was explanted, and one case suffered from facial nerve weakness. For the latter the integrity testing was inconclusive. Most device failures were of the brand Cochlear (*n* = 10, 62.5%). Two cases became non-users after re-implantation, one case was due to disappointing audiological results, the other case was due to persistent pain symptoms. The latter case was explanted a year and 8 months after re-implantation.

## 4 Discussion

The analysis of CT data of 192 ears of pediatric patients aged 0–17 years, showed that in the majority of the cases (79.7%, *n* = 153) it was not feasible to drill a bony well deep enough to lower the profile of the housing. The temporal bone thickness has been studied previously for the safety of implanting various bone-anchoring devices. In most cases a thickness of at least 3 mm was found in patients of five years and older. Below the age of five, several patients had a thickness of less than 3 mm. However, these studies used either a fixed location on the skull where the measurement took place (32, 33) or searched randomly within the segmented temporal bone (34). In our study, the search for an optimal location was systematic and the ROI was defined based on surgical practices for cochlear implantation. Our analysis of the most optimal location in the ROI showed that the mean minimum bone thickness for the age group 0–4 years was 1.84 mm with a range of 0.07 mm to 2.93 mm (Figure 3). These data confirm the difficulty and even impossibility in this age group, of drilling a bony well that complies with the advised dimensions of CI manufacturers. This is due not only to the depth of the recess but also the surface area that needs to be drilled out in order to accommodate the implant housing. This surface area is larger for the current R/S devices than previous generations (28). The curvature of the skull and irregularity of the surface increase the probability of exposing the dura mater.

In the retrospective review of our pediatric cohort data, 344 implants were placed with a complication rate of 8.1% (*n* = 29) major and 48.3% (*n* = 227) minor complications. The tight pocket technique was more frequently applied in the major complication group of which origin is questionably relate to the specific implant fixation technique. The most frequent major complication was infection of the implant site (20.7%, *n* = 6). Also no apparent difference was found in the fixation subgroups regarding device failure.

Previous studies on CI implantation in infants and small children have advised a limited bony recess due to the thin cranial bone (11, 35). To avoid risks such as dura exposure, especially in very young children, alternative fixation techniques have been introduced. In 2009 Balkany et al. (28) first reported the minimally invasive subperiosteal tight pocket technique. Variations of this technique have since been applied in pediatric and adult cohorts reporting a low major complication rate of 0%–5.2% (11, 35–37). Jethanamest et al. (38) reported no device migration or any complications related to device migration using the subperiosteal tight pocket technique. Some surgeons prefer to create a shallow well to fixate the implant (11, 35).Our clinical data on the complication rates of the different fixation techniques were inconclusive. Although the tight pocket technique was used more frequently is the major complications subgroup than the general cohort, there was no apparent difference in the rate of R/S device related issues between fixation technique groups, such as R/S device migration, infection of the implant or electrode array migration or extrusion. Furthermore, all tight pocket cases in the major complications subgroup were also operated with the SMA technique. The sample size of the tight pocket subgroup was too small to perform a statistical significance analysis. A previous review on R/S device complications in adults reported no evidence of a difference for the different fixation techniques (39). To fill this knowledge gap we are doing more research on R/S device related complications by directly comparing the two fixation techniques (bony well vs. tight pocket) in a prospective, randomized controlled study design (40).

The differences found in our retrospective study regarding the cochlear approach subgroups, were noteworthy. The most frequently used technique was the MPTA technique (74.4%, *n* = 256) (Table 4). However, in the major complications group, the most frequently applied surgical technique was the SMA technique (58.6%, *n* = 17), contrary to the general cohort. These findings are in line with an older study that included part of our cohort (14). The minor complication rate has increased over the years which could be explained by the increase of children operated under 12 months age. We included 102 children (29.7%) operated under the age of 12 months, vs. 17.7% (*n* = 33) that were included previously. The high number of young children could also explain the high rate of minor complications in our cohort of 48.3% (*n* = 166), compared to the literature, that reports rates of 1.8%–16% (10, 11, 41–43). Infectious (minor) complications such as acute otitis media and mastoiditis are known to occur more frequently in children under the age of 12 months, and comprised 30.9% (*n* = 86) of the minor complications in our cohort (44, 45). The higher rate could also be due to a difference in classification of complications, or potential bias such as information bias or selection bias (30).

This study is also at risk of beforementioned biases due to the retrospective design. Chart reviews are often incomplete, as was the case in our study. Due to the retrospective, non-standardized design we miss data that the observer/surgeon/doctor did not report. There could be variability in identification of complications. Moreover, the majority of CI's implanted in our study were of the brand Cochlear (*n* = 307, 89.2%) and most were of the CI400 series or older. The older CI models have different dimensions (thicker profile) making the comparison of R/S device related complications between subgroups difficult. Limitations are also introduced by the use of an in-house designed algorithm. However the effect of these limitations are minimized thanks to the large population size, detailed follow up and clinical application of the used algorithm. It should be noted that the algorithm searched the most optimal location within a predetermined ROI, based on expert opinion which could vary depending on the CI surgeon.

## 5 Conclusion

Based on the results of this study we would recommend the MPTA surgical technique over the SMA technique for cochlear approach. The results concerning the fixation techniques for the R/S device were inconclusive, but there is reason to question the current practices in pediatric patients of drilling out a bony well, especially in the 0–4 years age group. There is currently no evidence of a difference of the two surgical techniques regarding R/S migration and electrode array migration in adults (39). Further research is needed to validate complication differences in light of patients experiences (46). These outcomes are investigated in our ongoing randomized controlled trial, the results of which will be published in a peer-reviewed journal (40).

## Data Availability

The raw data supporting the conclusions of this article will be made available by the authors, without undue reservation.
